# 

**Published:** 1920-01-17

**Authors:** 


### THE HOSPITAL

**The Modern Newspaper of Administrative Medicine and Institutional Life.**

Telegraphic Address : OSPEDALE, RAND, LONDON. Telephone Number: 2734 GERRARD

No. 1754, Vol. LXVII. | Saturday, January 17th, 1920. | PRICE TWOPENCE

